# Subtype Differences in Pre-Coupling of Muscarinic Acetylcholine Receptors

**DOI:** 10.1371/journal.pone.0027732

**Published:** 2011-11-16

**Authors:** Jan Jakubík, Helena Janíčková, Alena Randáková, Esam E. El-Fakahany, Vladimír Doležal

**Affiliations:** 1 Department of Neurochemistry, Institute of Physiology Academy of Sciences of the Czech Republic v.v.i., Prague, Czech Republic; 2 Department of Experimental and Clinical Pharmacology, University of Minnesota College of Pharmacy, Minneapolis, Minnesota, United States of America; University of Oldenburg, Germany

## Abstract

Based on the kinetics of interaction between a receptor and G-protein, a myriad of possibilities may result. Two extreme cases are represented by: 1/Collision coupling, where an agonist binds to the free receptor and then the agonist-receptor complex “collides” with the free G-protein. 2/Pre-coupling, where stable receptor/G-protein complexes exist in the absence of agonist. Pre-coupling plays an important role in the kinetics of signal transduction. Odd-numbered muscarinic acetylcholine receptors preferentially couple to G_q/11_, while even-numbered receptors prefer coupling to G_i/o_. We analyzed the coupling status of the various subtypes of muscarinic receptors with preferential and non-preferential G-proteins. The magnitude of receptor-G-protein coupling was determined by the proportion of receptors existing in the agonist high-affinity binding conformation. Antibodies directed against the C-terminus of the α-subunits of the individual G-proteins were used to interfere with receptor-G-protein coupling. Effects of mutations and expression level on receptor-G-protein coupling were also investigated. Tested agonists displayed biphasic competition curves with the antagonist [^3^H]-N-methylscopolamine. Antibodies directed against the C-terminus of the α-subunits of the preferential G-protein decreased the proportion of high-affinity sites, and mutations at the receptor-G-protein interface abolished agonist high-affinity binding. In contrast, mutations that prevent receptor activation had no effect. Expression level of preferential G-proteins had no effect on pre-coupling to non-preferential G-proteins. Our data show that all subtypes of muscarinic receptors pre-couple with their preferential classes of G-proteins, but only M_1_ and M_3_ receptors also pre-couple with non-preferential G_i/o_ G-proteins. Pre-coupling is not dependent on agonist efficacy nor on receptor activation. The ultimate mode of coupling is therefore dictated by a combination of the receptor subtype and the class of G-protein.

## Introduction

G-protein coupled receptors (GPCR) represent the largest family of receptors, with more than 900 encoding genes [1]. They process and transduce a multitude of signals elicited by hormones, neurotransmitter and odorants and are thus involved in a very wide array of physiological and pathological processes. This makes this class of receptors a major pharmacological target for drug development [2].

Agonist-stimulated GPCRs in turn activate heterotrimeric GTP-binding proteins (G-proteins) that activate various signaling pathways. Two distinctive types of interaction between a receptor and G-protein exist: collision coupling and pre-coupling. In the former case, an agonist binds to the free receptor, activates it and then the receptor with bound agonist “collides” with free G-protein and activates it. In the latter case, stable receptor-G-protein complexes exist in the absence of agonist, agonist binds to this complex, induces change in the receptor conformation that leads to G-protein activation and dissociation of the complex [3]. It should, however, be noted that the distinction between collision coupling and pre-coupling is rather a matter of kinetics of receptor-G-protein interaction, activation state and receptor to G-protein stoichiometry [4]. Additional modes of interaction intermediate between pure collision coupling and pre-coupling, like transient receptor to G-protein complexing (“dynamic scaffolding”), have been observed [5].

There is accumulating evidence for both collision coupling and pre-coupling of GPCRs. Interestingly, coimmunoprecipitation studies showed pre-coupling of α_2A_-adrenergic receptors [6] with G_i/o_ G-proteins and β_2_-adrenergic receptors with G_s/olf_ G-proteins [7]. In contrast, rapid collision coupling of G-proteins with α_2A_-adrenergic receptors has been demonstrated in resonance energy transfer studies [8] and with β_2_-adrenergic receptors in living cell imaging studies [9]. Overall, current data on GPCR coupling suggest that the mode of receptor to G-protein coupling may differ depending on the receptor type, cell type and membrane composition [3], [10]. Thus, understanding the dynamic behavior of GPCR systems including receptor-G-protein coupling is important in discovery and development of more organ-specific drugs.

Muscarinic acetylcholine receptors are GPCRs present at synapses of the central and peripheral nervous systems but also exist in non-innervated cells and tissues. There are five subtypes of muscarinic receptors encoded by distinct genes without splicing variants [11]. Development of selective ligands for muscarinic receptors thus represents an enormous challenge due to their omnipresence, with only a few types of tissues being endowed by a single or predominant subtype of these receptors. So far very little is known about the nature of coupling of muscarinic receptors to G-proteins [12]. We have demonstrated that the M_2_ receptor can directly activate all three classes of G-proteins [13], and that it probably pre-couple to G_i/o_ but not to G_s/olf_ G-proteins [14]. To further clarify the mechanisms of muscarinic receptor subtypes signaling we analyzed the mode of coupling of M_1_ through M_4_ muscarinic receptors with G_i/o_, G_s/olf_ and G_q/11_ G-proteins in membranes from Chinese hamster ovary cells expressing individual receptor subtypes. We show that while M_1_ and M_3_ receptors pre-couple both with their preferential G_q/11_ and non-preferential G_i/o_ G-proteins, M_2_ and M_4_ receptors pre-couple only to preferential G_i/o_ G-proteins.

## Results

### Stimulation of [^35^S]GTPγS binding to G_i/o_, G_s/olf_ and G_q/11_ G-proteins

**Figure 1:**
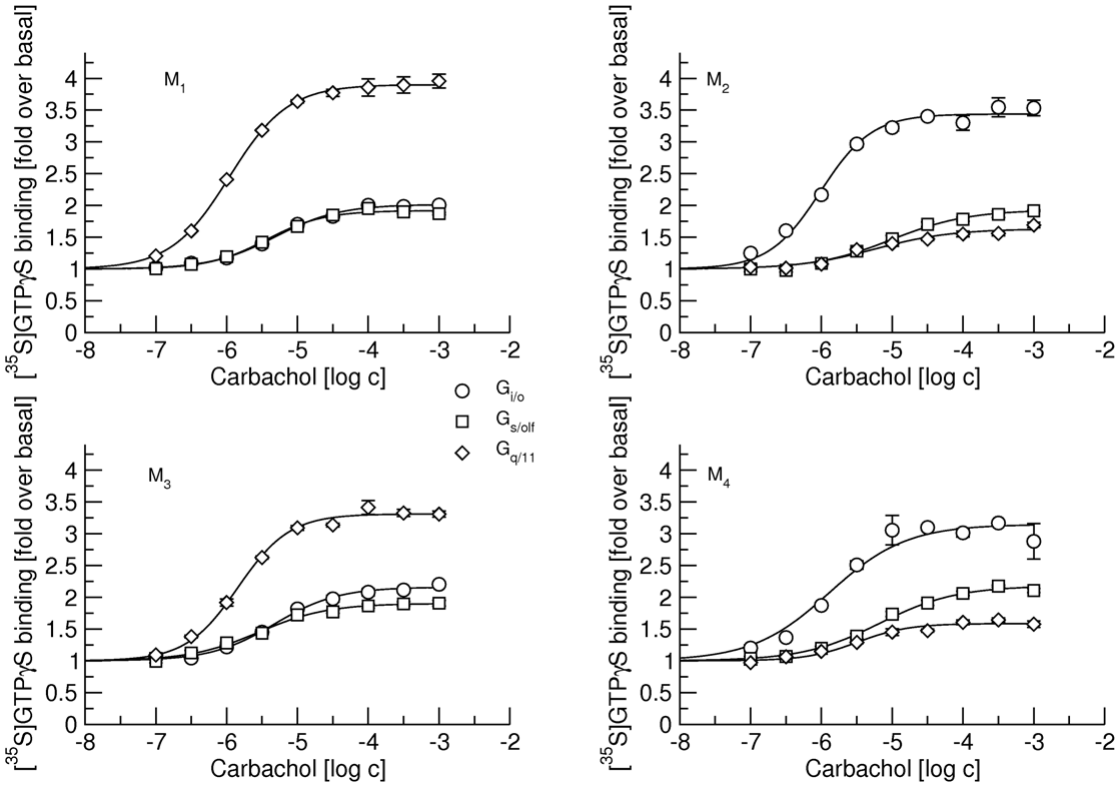
Stimulation of [^35^S]GTPγS binding by carbachol. [^35^S]GTPγS binding to G_i/o_ (circles), G_s/olf_ (squares) and G_q/11_ G-proteins (diamonds) via M_1_ (upper left), M_2_ (upper right), M_3_ (lower left) and M_4_ (lower right) receptors by increasing concentrations (abscissa, log M) of carbachol is expressed as fold over basal (ordinate). Data are means ± S.E.M of values from 3 experiments performed in quadruplicates. Curves were fitted using equation 2 and results of fits are shown in Table 1.

Membranes from CHO cells containing from 1.4 to 2.5 fmol of M_1_ through M_4_ muscarinic receptors per mg of protein were exposed to carbachol in concentrations ranging from 0.1 µM to 1 mM and binding of [^35^S]GTPγS to G-protein classes was determined using a scintillation proximity assay (SPA) (Fig. 1). Carbachol stimulated [^35^S]GTPγS binding to all three major classes of G-proteins via all four receptors, with highest potency (EC_50_ about 1 µM) and efficacy (more than 3-fold increase over basal) for preferential G-proteins (G_q/11_ for M_1_ and M_3_ and G_i/o_ for M_2_ and M_4_ receptors) (Table 1). The potency of carbachol in stimulating [^35^S]GTPγS binding to non-preferential G-proteins was 2- (M_3_ G_s_/_olf_) to 10-fold (M_2_ G_s/olf_) lower than to preferential G-proteins.

**Table 1:** Stimulation of [^35^S]GTPγS binding by carbachol to G_i/o_, G_s/olf_ and G_q/11_ subtypes of G-proteins via M_1_ through M_4_ receptors.

	G_i/o_		G_s/olf_		G_q/11_	
	pEC_50_	E_MAX_	pEC_50_	E_MAX_	pEC_50_	E_MAX_
M_1_	5.31±0.05	2.01±0.05	5.44±0.05	1.92±0.05	5.96±0.05	3.90±0.08
M_2_	6.01±0.06	3.44±0.07	5.01±0.06	1.93±0.05	5.24±0.02	1.63 ±0.02
M_3_	5.32±0.05	2.16±0.06	5.54±0.05	2.16±0.05	5.83±0.04	3.31±0.06
M_4_	5.89±0.05	3.15±0.08	5.18±0.06	2.18±0.06	5.49±0.05	1.59±0.04

Data are means ± S.E.M. From 3 experiments performed in quadruplicates. E_MAX_ is expressed as fold increase of basal binding.

### Competition of carbachol with [^3^H]NMS binding at M_1_ through M_4_ receptors

**Figure 2:**
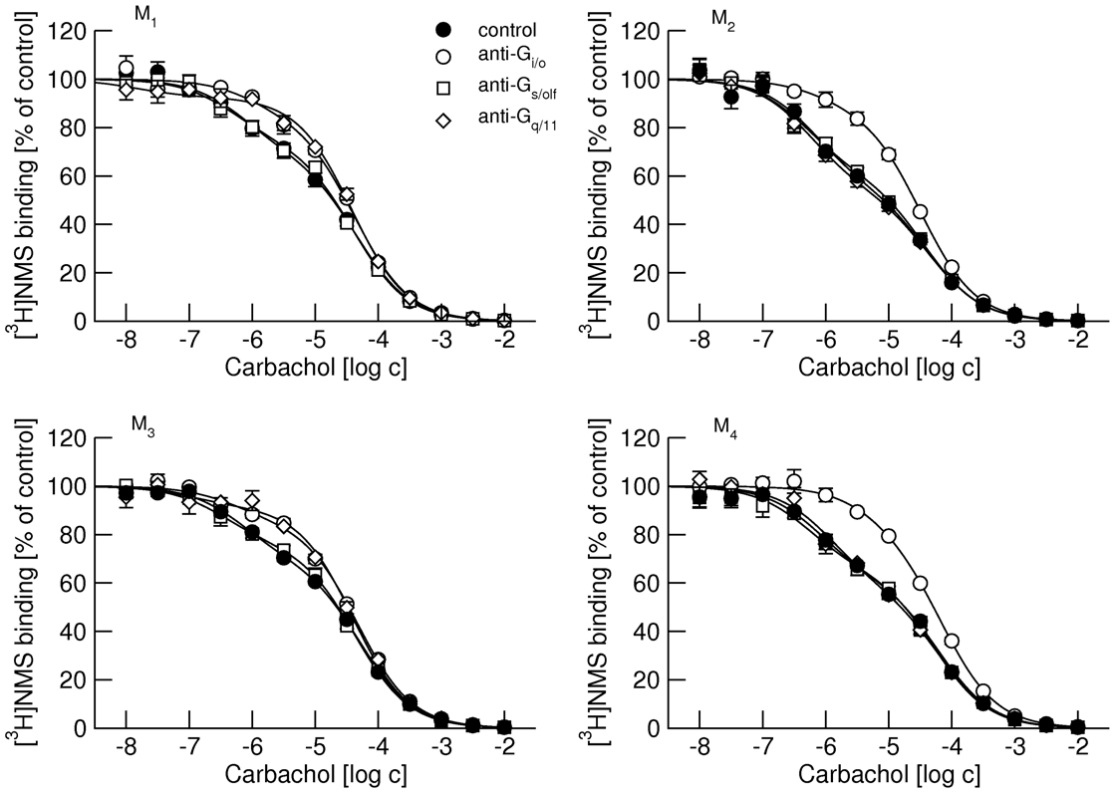
Effects of anti-G-protein antibodies on competition between carbachol and [^3^H]NMS binding at M_1_ to M_4_ receptors. Binding of 1 nM [^3^H]NMS to membranes from CHO cells expressing M_1_ (upper left), M_2_ (upper right), M_3_ (lower left) and M_4_ (lower right) receptors in the presence of increasing concentrations (abscissa, log M) of carbachol is expressed as per cent of control binding in the absence of carbachol. Filled circles, control binding in the absence of antibodies. Open symbols, binding in the presence of anti-G_i/o_ (circles), anti-G_s/olf_ (squares), and anti-G_q/11_ (diamonds) antibodies. Data are means ± S.E.M of values from 3 experiments performed in quadruplicates. Curves were fitted using equation 3 and results of fits are shown in the Table 2.

Binding of the tritiated antagonist N-metylscopolamine ([^3^H]NMS) in the presence of agonist carbachol concentrations ranging from 10 nM to 10 mM (Fig. 2) was best described by competition for two sites (Eq. 3) at all four receptor subtypes. The equilibrium inhibition constant (K_I_) of carbachol was similar among receptor subtypes, both for high and low affinity sites (Table 2). At M_1_ and M_3_ receptors that preferentially couple to G_q/11_ G-proteins carbachol displayed more low affinity binding sites than at M_2_ and M_4_ receptors that preferentially couple to G_i/o_ G-proteins. In some cases preincubation of membranes with antibodies directed against the C-termini of α-subunits of individual classes of G-proteins led to an increase in the proportion of low affinity sites. The proportion of low affinity sites was increased by anti-G_i/o_ and anti-G_q/11_ antibodies at M_1_ and M_3_ receptors but only by anti-G_i/o_ antibodies at M_2_ and M_4_ receptors. The anti-G_s/olf_ antibody did not change the proportion of low affinity sites at any receptor subtype. None of the antibodies affected K_I_ of either the low or high affinity sites.

**Table 2:** Effects of IgG antibodies directed against the α-subunits of individual subtypes of G-proteins on binding parameters of carbachol in membranes of CHO cells expressing individual subtypes of muscarinic receptors.

		control	anti-G_i/o_	anti-G_s/olf_	anti-G_q/11_
M_1_	pK_i high_	7.01±0.08	6.93±0.09	7.09±0.08	7.14±0.08
	pK_i low_	5.21±0.07	5.25±0.06	5.27±0.07	5.30±0.07
	f _low_	69±7	84±8*	74±7	92±8*
M_2_	pK_i high_	6.81±0.08	6.70±0.07	6.93±0.08	6.88±0.07
	pK_i low_	5.01±0.07	5.13±0.07	5.06±0.07	5.01±0.08
	f _low_	56±8	89±8*	61±9	54±9
M_3_	pK_i high_	7.03±0.08	7.15±0.09	7.23±0.08	7.14±0.09
	pK_i low_	5.15±0.09	5.20±0.09	5.23±0.08	5.27±0.09
	f _low_	72±7	89±9*	76±7	92±8*
M_4_	pK_i high_	6.91±0.09	7.03±0.08	7.09±0.09	6.94±0.08
	pK_i low_	5.01±0.09	4.95±0.08	5.08±0.09	5.00 ±0.08
	f _low_	62±7	82±8*	66±7	58±7

Data are means ± S.E.M. From 3 experiments performed in quadruplicates. f_low_, fraction of low-affinity sites in percent;

*, significantly different from control by t-test (P<0.05).

### Competition of agonists with [^3^H]NMS binding at M_1_ and M_2_ receptors

**Figure 3:**
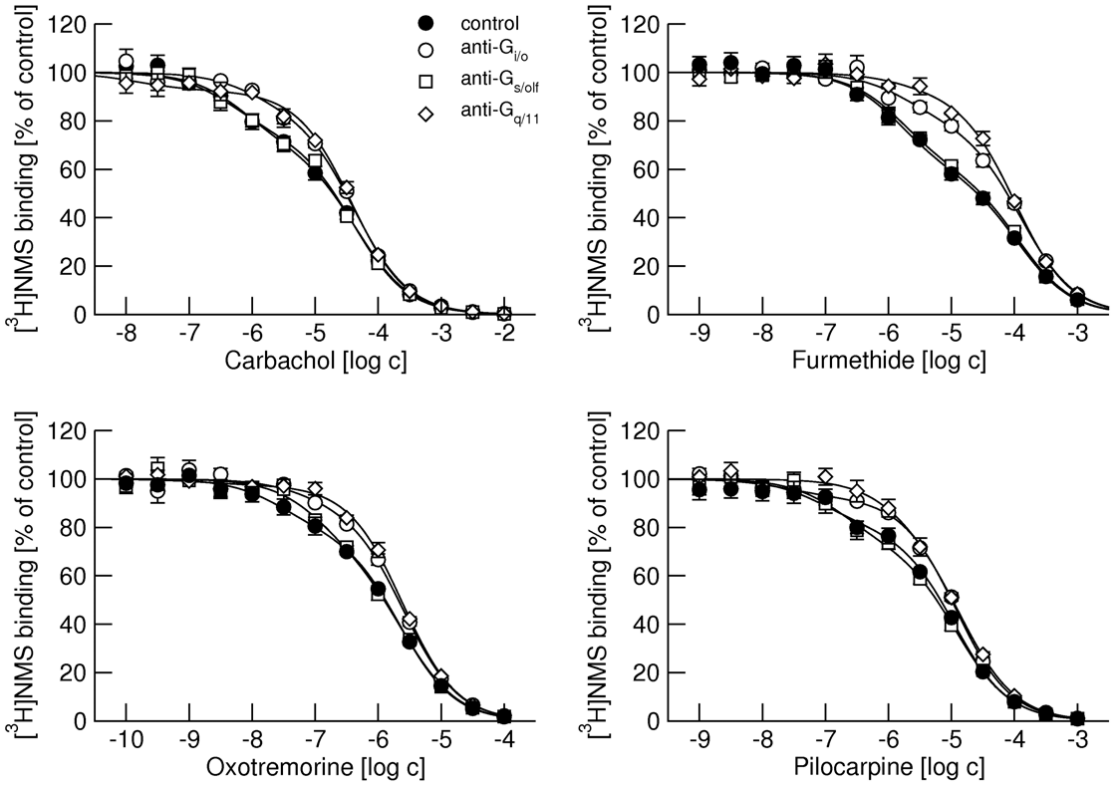
Effects of anti-G-protein antibodies on competition between different agonists and [^3^H]NMS binding at M_1_ receptors. Binding of 1 nM [^3^H]NMS to membranes from CHO cells expressing M_1_ receptors in the presence of increasing concentrations (abscissa, log M) of the agonists carbachol (upper left), furmethide (upper right), oxotremorine (lower left) and pilocarpine (lower right) is expressed as per cent of control binding in the absence of agonist. Filled circles, control binding in the absence of antibodies. Open symbols, binding in the presence of anti-G_i/o_ (circles), anti-G_s/olf_ (squares) and anti-G_q/11_ (diamonds) antibodies. Data are means ± S.E.M of values from 3 experiments performed in quadruplicates. Curves were fitted using equation 3 and results of fits are shown in Table 3.

All tested agonists at M_1_ receptors (carbachol, furmethide, oxotremorine, and pilocarpine) bound to two binding sites (Fig. 3, full circles). Although they bound with different affinities they recognized the same proportion of low-affinity sites (Table 3). Anti-G_i/o_ and anti-G_q/11_ antibodies increased the proportion of low affinity sites to a comparable extent for all tested agonists (Fig. 3, open circles and open diamonds). The anti-G_s/olf_ antibody did not change the proportion of the low-affinity binding sites for any of the agonists tested (Fig. 3, open squares). None of the antibodies affected K_I_ values.

**Table 3:** Effects of IgG antibodies directed against α-subunits of individual subtypes of G-proteins on binding parameters of different muscarinic agonists in membranes of M_1_ CHO cells.

		Control	anti-G_i/o_	anti-G_s/olf_	anti-G_q/1_
carbachol	pK_i high_	7.01±0.08	6.93±0.09	7.09±0.08	7.14±0.08
	pK_i low_	5.21±0.07	5.25±0.06	5.27±0.07	5.30±0.07
	f _low_	69±7	86±8*	74±7	92±8*
furmethide	pK_i high_	6.70±0.07	6.59±0.08	6.66±0.07	6.63±0.08
	pK_i low_	4.82±0.07	4.78±0.07	4.81±0.07	4.87±0.07
	f _low_	62±7	84±8*	62±6	92±8*
oxotremorine	pK_i high_	8.12±0.08	8.04±0.8	7.96±0.06	8.14±0.08
	pK_i low_	6.53±0.06	6.47±0.07	6.46±0.06	6.49±0.06
	f _low_	69±6	90±8*	69±6	95±5*
pilocarpine	pK_i high_	7.72±0.07	7.64±0.07	7.61±0.07	7.66±0.07
	pK_i low_	5.84±0.06	5.81±0.06	5.80±0.06	5.71±0.06
	f _low_	75±7	92±8*	73±7	90±8*

Data are means ± S.E.M. From 3 experiments performed in quadruplicates. f_low_, fraction of low-affinity sites in percent;

*, significantly different from control by t-test (P<0.05).

**Figure 4:**
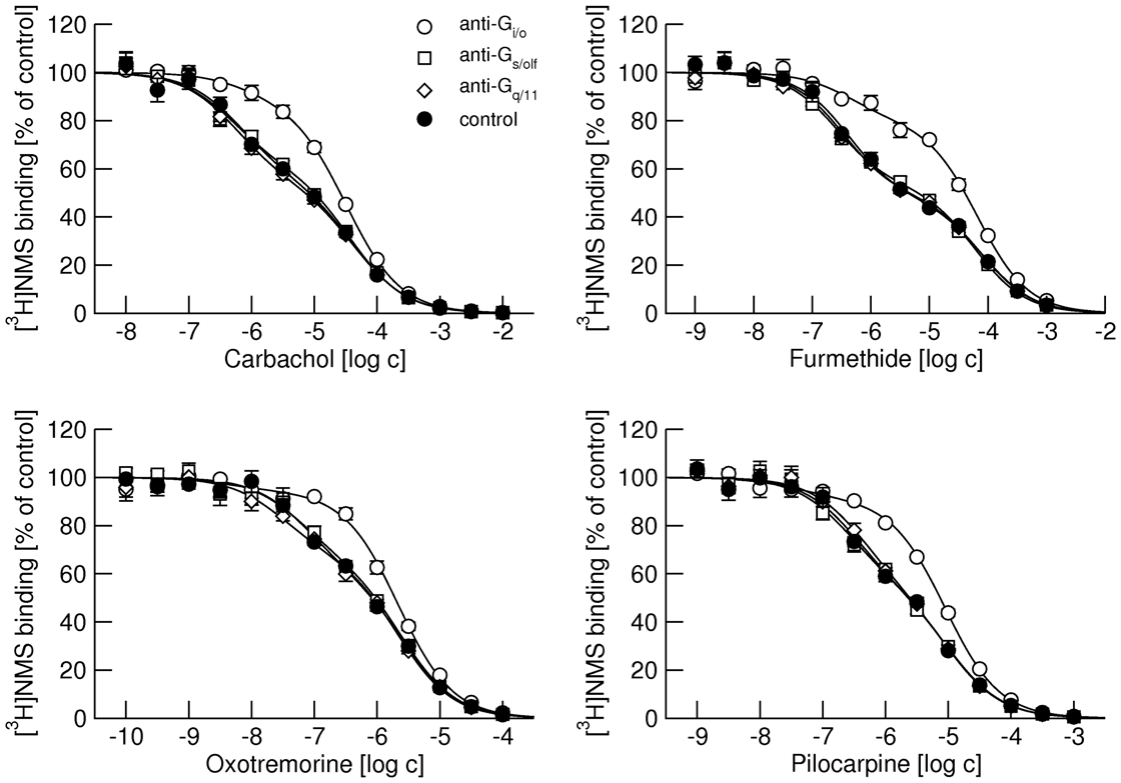
Effects of anti-G-protein antibodies on competition between different agonists and [^3^H]NMS binding at M_2_ receptors. Binding of 1 nM [^3^H]NMS to membranes from CHO cells expressing M_2_ receptors in the presence of increasing concentrations (abscissa, log M) of the agonists carbachol (upper left), furmethide (upper right), oxotremorine (lower left) and pilocarpine (lower right) is expressed as per cent of control binding in the absence of agonist. Filled circles, control binding in the absence of antibodies. Open symbols, binding in the presence of anti-G_i/o_ (circles), anti-G_s/olf_ (squares) and anti-G_q/11_ (diamonds) antibodies. Data are means ± S.E.M of values from 3 experiments performed in quadruplicates. Curves were fitted using equation 3 and results of fits are shown in Table 4.

Similarly, all tested agonists bound to two binding sites at M_2_ receptors (Fig. 4, full circles). As in the case of the M_1_ receptor they bound with different affinities but they recognized the same proportion of low-affinity sites (Table 4). Similar to carbachol, the proportion of low-affinity sites was lower at M_2_ than at M_1_ receptors for all tested agonists (Table 4 vs. Table 3) and only the anti-G_i/o_ antibody increased the proportion of low affinity sites (Fig. 4, open circles). The anti-G_s/olf_ and anti G_q/11_ antibodies did not change either the proportion of low-affinity binding sites or K_I_s for any of the agonists tested (Fig. 4, open squares and open diamonds).

**Table 4:** Effects of IgG antibodies directed against α-subunits of individual subtypes of G-proteins on binding parameters of different muscarinic agonists in membranes of M_2_ CHO cells.

		Control	anti-G_i/o_	anti-G_s/olf_	anti-G_q/1_
carbachol	pK_i high_	6.81±0.08	6.70±0.07	6.93±0.08	6.88±0.07
	pK_i low_	5.01±0.07	5.13±0.07	5.06±0.07	5.01±0.08
	f _low_	56±8	89±8*	61±9	54±9
furmethide	pK_i high_	6.99±0.08	6.98±0.07	7.19±0.07	7.08±0.08
	pK_i low_	4.70±0.08	4.79±0.07	4.84±0.08	4.73±0.07
	f _low_	48±8	80±7*	49±8	53±9
oxotremorine	pK_i high_	7.74±0.09	7.76±0.08	7.83±0.08	7.93±0.08
	pK_i low_	6.17±0.08	5.78±0.08	6.22±0.09	6.27±0.08
	f _low_	60±8	84±7*	65±8	61±7
pilocarpine	pK_i high_	7.10±0.09	7.05±0.11	7.22±0.09	7.00±0.10
	pK_i low_	5.58±0.09	5.67±0.09	5.62±0.09	5.60±0.09
	f _low_	53±10	76±11*	53±10	57±11

Data are means ± S.E.M. From 3 experiments performed in quadruplicates. f_low_, fraction of low-affinity sites in percent;

*, significantly different from control by t-test (P<0.05).

### Effects of mutations of M_1_ receptors that affect receptor activation

To further investigate the role of receptor activation in receptor-G-protein pre-coupling we prepared cell lines expressing mutant M_1_ receptor with mutations known to interfere with receptor signaling. Mutation of aspartate 71 in the middle of the second transmembrane domain to asparagine (D71N) has been shown to abolish receptor activation [15]. Mutation of aspartate 122 in the conserved E/DRY-motif at the intracellular edge of the third transmembrane domain to asparagine (D122N) has been shown to reduce the potency of muscarinic agonists [16]. Opsin arginine in the conserved E/DRY-motif at the intracellular edge of the third transmembrane domain of has been shown to directly interact with the C-terminal cysteine of the α-subunit of G-protein [16]. At M_1_ muscarinic receptors mutation of corresponding arginine 123 asparagine (R123N) blocks activation of G-proteins [17]. The appropriate control CHO cell line expressing the wild-type receptor was also generated using the same expression vector. Expression levels of receptor mutants (0.42 to 0.87 pmol per mg of protein) were the same as expression level of the wild-type receptor (0.63 to 0.71 pmol per mg of protein).

**Figure 5:**
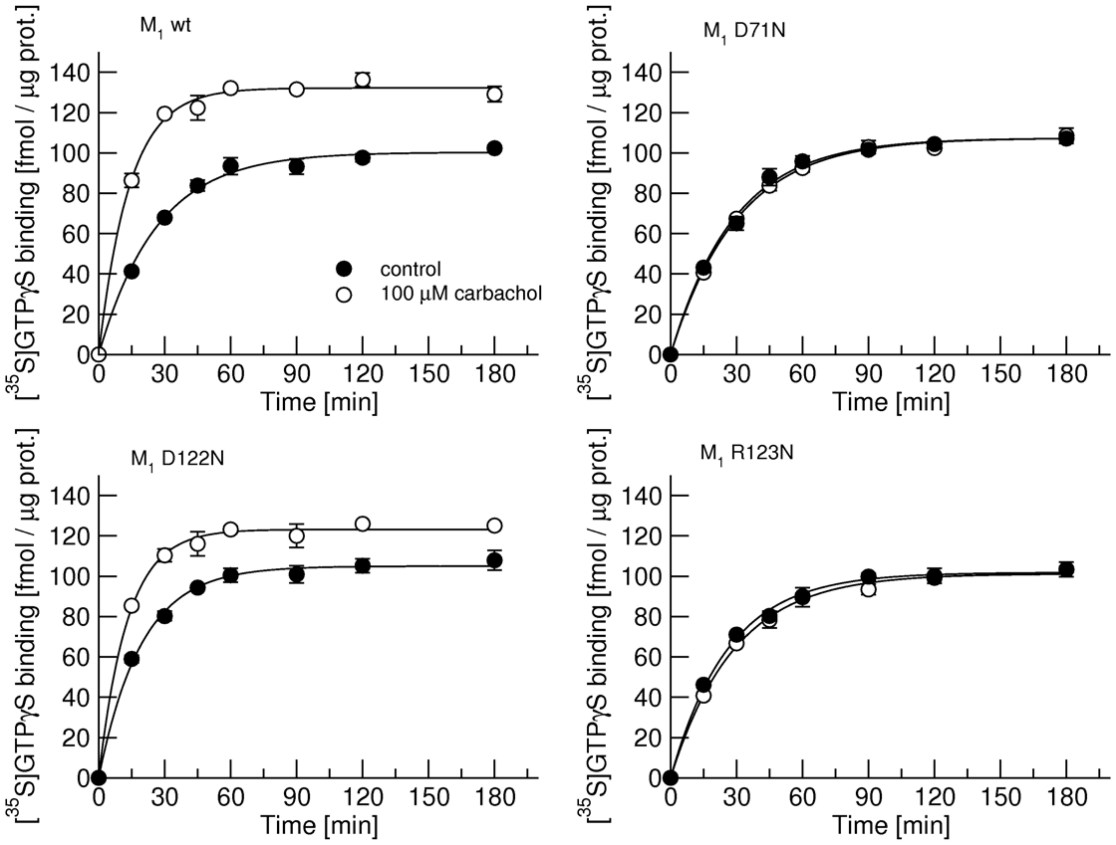
Effects of point mutations of the M_1_ receptor on carbachol-induced stimulation of of [^35^S]GTPγS. Binding of 0.5 nM [^35^S]GTPγS to all G-proteins in the presence of 50 µM GDP was measured in membranes from newly prepared CHO cell lines expressing either wild-type (M_1_ wt) or mutant (D71N, D122N, R123N) M_1_ receptors in the absence (full circles) or in the presence (open circles) of 100 µM carbachol. Data are means ± S.E.M of values from 3 experiments performed in quadruplicates. Curves were fitted using equation 4 and results of fits are shown in Table 5.

Association of 0.5 nM [^35^S]GTPγS with membranes from the newly prepared CHO cell line expressing M_1_ receptors occurred with observed association rate k_obs_ =  0.036 min^-1^ (Fig. 5 upper left, full circles, and Table 5). One hundred µM carbachol (Fig. 5, open circles) accelerated association of [^35^S]GTPγS two-times and increased equilibrium binding (B_eq_) by one third. Mutations D71N (Fig. 5 upper right) and R123N (Fig. 5 lower right) did not change basal (in the absence of carbachol) association of [^35^S]GTPγS but they both abolished acceleration induced by carbachol. Mutation D122N accelerated basal association of [^35^S]GTPγS by 50%. One hundred µM carbachol further accelerated association of [^35^S]GTPγS. The rate of association as well as B_eq_ in the presence of carbachol at R123N receptors was the same as at control (M_1_ wt) (Fig. 5 and Table 5).

**Table 5:** Rates of basal and carbachol-stimulated association of [^35^S]GTPγS in CHO membranes expressing wild type and mutant M_1_ receptors.

		M_1_ wt	D71N	D122N	R123N
control	k_obs_ [min^-1^]	0.036±0.007	0.034±0.007	0.054±0.005#	0.038±0.007
	B_eq_[fmol/∶g prot.]	101±6	107±12	103±8	105±6
+100 µM carbachol	k_obs_ [min^-1^]	0.075±0.006*	0.033±0.006#	0.077±0.005*	0.036±0.007#
	B_eq_[fmol/µg prot.]	133±8*	105±9#	126±6*	100±5#

Data are means ± S.E.M. From 3 experiments performed in quadruplicates.

*, Significantly different from control;

# , significantly different from wild type (M_1_ wt), t-test (P<0.05).

**Figure 6:**
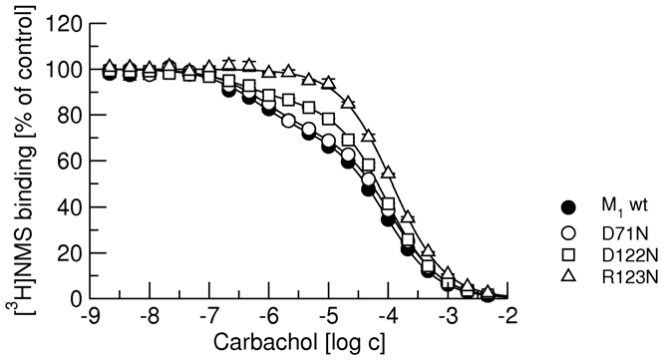
Effects of point mutations of the M_1_ receptor on competition between carbachol and [^3^H]NMS binding. Binding of 1 nM [^3^H]NMS to membranes from newly prepared CHO cell lines expressing wild type and mutant M_1_ receptors in the presence of increasing concentrations carbachol is expressed as per cent of control binding in the absence of agonist. Filled circles, binding to wild-type M_1_ receptors. Open symbols, binding to D71N (circles), D122N (squares) and R123N (triangles) mutatant M_1_ receptors. Data are means ± S.E.M of values from 3 experiments performed in quadruplicates. Curves were fitted using equation 3 and results of fits are shown in Table 6.

On the newly prepared cell line expressing M_1_ wt receptors carbachol displayed binding to two binding sites in competition with [^3^H]NMS with the same proportion of low affinity sites and similar affinities (Fig. 6, full circles) as in Fig. 2. While mutation D71N did not change the binding parameters of carbachol, mutation D122N brought about an increase in low affinity sites and mutation R123N completely abolished high-affinity binding (Fig. 6 and Table 6).

**Table 6:** Effects of single amino acid mutations of M_1_ receptor on the binding parameters of carbachol.

	M_1_ control	D71N	D122N	R123N
pK_i high_	7.13±0.08	7.01±0.08	7.07±0.09	
pK_i low_	4.91±0.06	4.85±0.05	4.92±0.05	4.80±0.07
f _low_	72±6	74±7	86±7*	99±1*

Data are means ± S.E.M. From 3 experiments performed in quadruplicates. f_low_, fraction of low-affinity sites in percent;

*, significantly different from control by t-test (P<0.05).

### Effects of attenuation of expression of G_i/o_ G-proteins in M_2_-CHO cells

**Figure 7:**
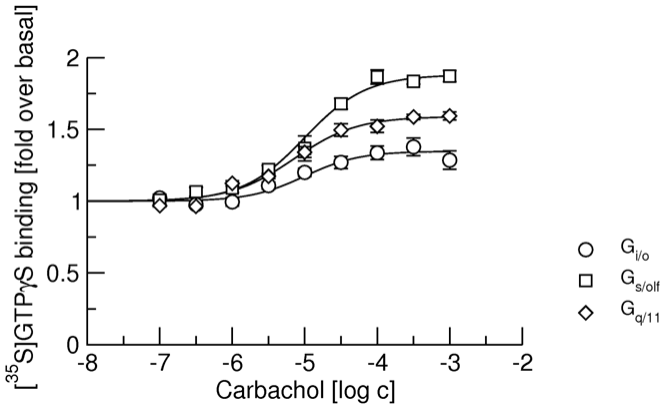
Stimulation of [^35^S]GTPγS binding by carbachol at the M_2_ receptor after suppression of expression of G_i/o_ G-proteins. M_2_ receptor-mediated stimulation of [^35^S]GTPγS binding to G_i/o_ (circles), G_s/olf_ (squares) and G_q/11_ G-proteins (diamonds) after suppression of expression of G_i/o_ G-proteins by siRNA was stimulated by increasing concentrations of carbachol (abscissa, log M). Response is expressed as fold over basal (ordinate). Data are means ± S.E.M of values from 3 experiments performed in quadruplicates. Curves were fitted using equation 2 and results of fits are shown in Table 5.

Total binding capacity of (saturating) 500 nM [^35^S]GTPγS in control M_2_-CHO membranes (in the absence of GDP) showed prevalence of G_i/o_ G-proteins over G_s/olf_ and G_q/11_ (37.5±3.9, 22.0±2.3 and 25.4±2.8 pmol/mg prot., respectively; mean ± S.E.M., n = 3). Treatment of M_2_-CHO cells with siRNA directed to G_i/o_ G-proteins resulted in more than a 70% decrease in the [^35^S]GTPγS binding capacity of G_i/o_ (10.1±1.8 pmol/mg prot.; mean ± S.E.M., n = 3) without a change in the binding capacity of G_s/olf_ and G_q/11_ G-proteins (24.1±2.2, 23.8±2.5 pmol/mg prot., respectively; mean ± S.E.M., n = 3). This treatment resulted in a 10-fold decrease in the potency of carbachol in stimulation of [^35^S]GTPγS binding to G_i/o_ G-proteins (Fig. 7 vs. Fig. 1 upper right, open circles; Table 7 vs. Table 1) and decreased its efficacy more than 5-times. The efficacy of carbachol in stimulation of [^35^S]GTPγS binding to G_s/olf_ or G_q/11_ G-proteins was unchanged while its potency increased about 3-times in both cases.

**Table 7:** Stimulation of [^35^S]GTPγS binding by carbachol via M_2_ receptors to G_i/o_, G_s/olf_ and G_q/11_ subtypes of G-proteins in membranes with reduced expression of the G_i/o_ subclass of G-proteins.

	pEC_50_	E_MAX_
G_i/o_	5.01±0.06	1.43±0.08
G_s/olf_	5.54±0.05	1.82±0.06
G_q/11_	5.65±0.05	1.63±0.04

Data are means ± S.E.M. From 3 experiments performed in quadruplicates. E_MAX_ is expressed as fold increase of basal binding.

**Figure 8:**
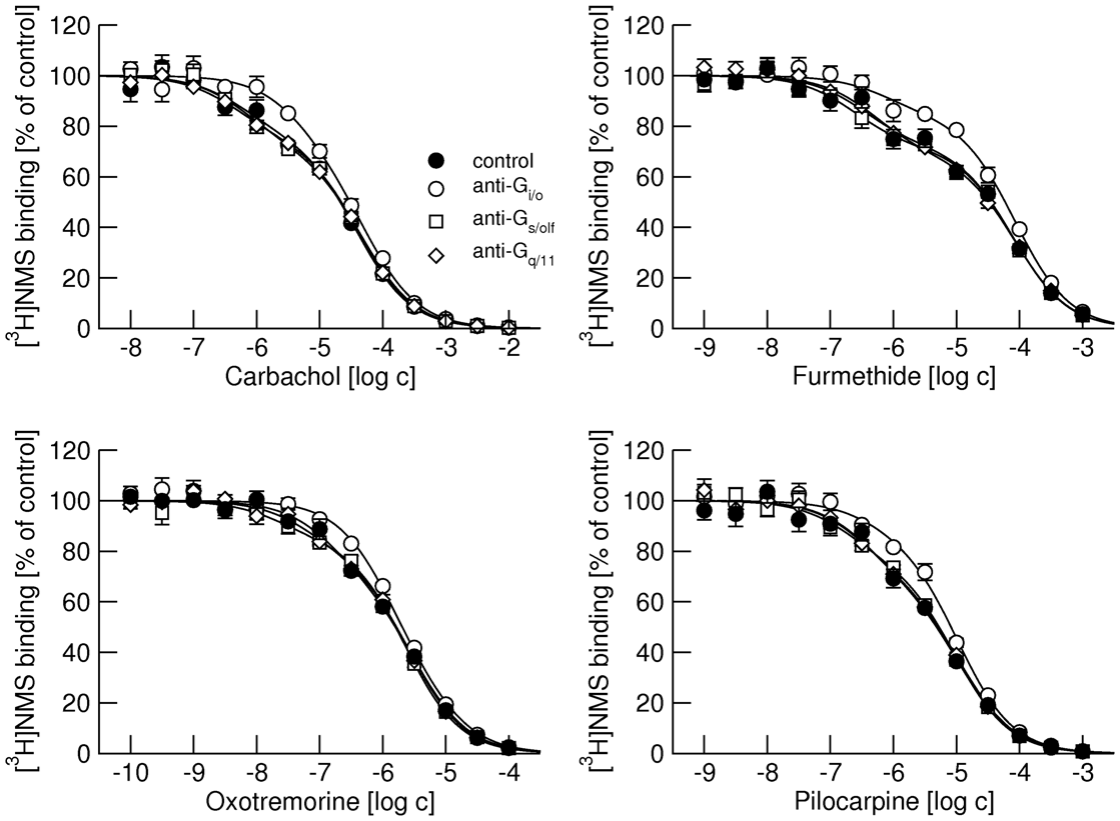
Effects of anti-G-protein antibodies on competition between agonists and [^3^H]NMS binding at M_2_ receptors after suppression of G_i/o_ G-proteins expression. Binding of 1 nM [^3^H]NMS to membranes from CHO cells expressing M_2_ receptors after suppression of expression of G_i/o_ G-proteins by siRNA was determined in the presence of increasing concentrations (abscissa, log M) of the agonists carbachol (upper left), furmethide (upper right), oxotremorine (lower left), and pilocarpine (lower right). Binding is expressed as per cent of control binding in the absence of agonist. Filled circles, control binding in the absence of antibodies. Open symbols, binding in the presence of anti-G_i/o_ (circles), anti-G_s/olf_ (squares) and anti-G_q/11_ (diamonds) antibodies. Data are means ± S.E.M of values from 3 experiments performed in quadruplicates. Curves were fitted using equation 3 and results of fits are shown in Table 6.

Based on competition binding of agonists and [^3^H]NMS (Fig. 8; Table 8), attenuation of G_i/o_ expression led to an increase in the proportion of low-affinity sites for all tested agonists (see controls in Table 4 and Table 8) without change in K_I_ values. The anti-G_i/o_ antibody further increased the proportion of low-affinity sites in G_i/o_ G-proteins-depleted membranes only for the full agonists carbachol and furmethide. In contrast to control M_2_-CHO cells, the proportion of low-affinity sites of the partial agonists oxotremorine and pilocarpine in G_i/o_ G-proteins-depleted membranes was not changed by the anti-G_i/o_ antibody. Similar to untreated M_2_-CHO cells, the anti-G_s/olf_ and anti-G_q/11_ antibodies had no effect on either the proportion of low affinity sites or K_I_ values in membranes with attenuated expression of G_i/o_ G-proteins.

**Table 8:** Effects of IgG antibodies directed against α-subunits of individual subtypes of G-proteins on binding parameters of muscarinic agonists in membranes of M_2_ CHO cells with reduced expression of G_i/o_ G-proteins by siRNA.

		control	anti-G_i/o_	anti-G_s/olf_	anti-G_q/11_
carbachol	pK_i high_	6.85±0.08	6.89±0.07	6.94±0.08	6.77±0.07
	pK_i low_	5.03±0.07	4.88±0.07	4.98±0.07	4.95±0.08
	f _low_	73±8	90±8*	75±9	72±9
furmethide	pK_i high_	7.07±0.08	6.72±0.07	7.91±0.07	6.97±0.08
	pK_i low_	4.72±0.08	4.68±0.07	4.69±0.08	4.71±0.07
	f _low_	72±7	86±7*	69±8	72±9
oxotremorine	pK_i high_	7.55±0.09	7.54±0.08	7.76±0.08	7.63±0.08
	pK_i low_	6.09±0.08	6.15±0.08	6.17±0.09	6.25±0.08
	f _low_	78±8	89±7	84±8	79±7
pilocarpine	pK_i high_	7.00±0.09	6.91±0.11	7.06±0.09	7.31±0.10
	pK_i low_	5.55±0.09	5.58±0.09	5.54±0.09	5.62±0.09
	f _low_	72±11	87±12	76±10	73±11

Data are means ± S.E.M. From 3 experiments performed in quadruplicates. f_low_, fraction of low-affinity sites in percent;

*, significantly different from control by t-test (P<0.05).

## Discussion

Binding of an agonist to a G-protein-coupled receptor (GPCR) results in transforming the receptor to an active state that facilitates guanosine diphosphate (GDP) dissociation from the α-subunit of interacting heterotrimeric G-proteins and its exchange for guanosine trisphosphate (GTP) [18]. In principle there are many possible ways for receptor-G-protein interactions to take effect, with two extreme possibilities. In one scenario receptors and G-proteins diffuse freely within the plasma membrane, agonist binds to the free receptor that then randomly “collides” with G-proteins and activates them. Alternatively, receptors and G-proteins form stable complexes regardless of the receptor activation state and agonist binding, the agonist binds to this complex and induces conformational changes in the receptor protein that leads to G-protein activation and dissipation of the receptor-G-protein complex. We will refer to the former situation as “collision coupling” and the latter one as “pre-coupling” [3]. It should, however, be noted that even if receptors are partially pre-coupled to G-proteins, an agonist can also bind to free receptors and then “collide” with G-protein. Also the distinction between collision coupling and pre-coupling is rather a matter of kinetics of receptor-G-protein interaction and activation and receptor to G-protein stoichiometry [4]. Thus a myriad of possible ways for interaction among receptor, G-protein and agonist exist, e.g., transient receptor to G-protein complexing (“dynamic scaffolding”) [5]. Receptor G-protein pre-coupling plays an important role in signaling. It may accelerate kinetics of signal transduction. If receptor G-protein complexes pre-exist, instantaneous activation of G-protein takes place upon agonist binding to the receptor [4].

As shown repeatedly [10], [13], [19], [20] and also in Fig. 1, muscarinic acetylcholine receptors couple with all 3 major classes of G-proteins (G_i/o_, G_s/olf_ and G_q/11_). Our recent data show that at M_2_ receptors the agonist carbachol slows down the association of GDP with G_i/o_ but not G_s/olf_ G-proteins [14]. This finding may evidence the pre-existence of a receptor/G-protein complex prior to carbachol binding. Data thus suggest that muscarinic M_2_ receptors pre-couple to G_i/o_ but not to G_s/olf_ G-proteins. Alternatively, M_2_ receptors may precouple with G_s/olf_ but carbachol has no effect on GDP association. To exclude this possibility we analyzed in detail pre-coupling of all 3 major classes of G-proteins with M_2_ receptors and compared it with pre-coupling at other G_i/o_ preferring (M_4_) and G_q/11_ preferring (M_1_ and M_3_) muscarinic receptors.

At all receptor subtypes carbachol displays a “two site” binding curve with high affinity binding in the nanomolar range and low affinity binding in the micromolar range (Fig. 2, Table 2). According to the ternary complex model of GPCRs [21] agonists bind with high affinity to the receptor-G-protein complex and with low-affinity to receptors uncoupled from G-proteins. The interface of interaction between the receptor and G-protein consists of the intracellular edge of the third, fifth and sixth transmembrane domains and adjacent parts of the third and fourth intracellular loops of the receptor and the C-terminus of the G-protein α-subunit [16], [22]. Antibodies directed against the C-terminus of G-protein should prevent receptor-G-protein interaction (or break existing receptor G-protein complex) and lower the affinity of the receptor for agonists. Indeed, IgG antibodies directed against the C-terminus of the G_i/o_ class of G-proteins increased the fraction of low-affinity sites at all receptor subtypes including the G_i/o_ non-preferring M_1_ and M_3_ receptors. Similarly, IgG antibodies directed against the C-terminus of G_q/11_ class of G-proteins increased the fraction of low-affinity sites only at their preferring M_1_ and M_3_ receptors, but IgG antibodies directed against the C-terminus of G_s/olf_ class of G-proteins had no effect. Antibodies only changed the proportion of low-affinity sites without an effect on receptor affinity. Our data also show that all receptors pre-couple with their preferential G-proteins (M_1_ and M_3_ with G_q/11_ and M_2_ and M_4_ with G_i/o_) and that M_1_ and M_3_ receptors also pre-couple with non-preferential G_i/o_ G-proteins. In contrast, pre-coupling of G_s/olf_ G-proteins was not detected at any subtype of muscarinic receptors. In other words, the interaction between receptor and G_s/olf_ is so short-lived that cannot be detected by antibodies. This is in agreement with our kinetic measurements at G_s/olf_ and M_2_ receptors [14].

We tested binding of four structurally different agonists (carbachol, furmethide, oxotremorine and pilocarpine) that also differ in potency and efficacy in activating muscarinic receptors [14] and binding kinetics [23]. Importantly, all tested agonists recognize the same proportion of low-affinity binding sites (Figs 3 and 4; Tables 3 and 4). It is very unlikely that these agonists induce the same proportion of transient high-affinity states (in collision coupling, dynamic scaffolding or a similar scenario). Rather receptor G-protein complexes preexist prior to agonists binding and their proportion is given by stoichiometry of receptors and G-proteins. Moreover, the antibodies have the same effect on binding of all agonists, further excluding the role of agonists in the formation of receptor-G-protein complexes.

To further investigate the role of receptor activation in receptor-G-protein pre-coupling we prepared cell lines expressing mutant M_1_ receptor with mutations known to interfere with receptor signaling. Mutation of aspartate 71 in the middle of the second transmembrane domain to asparagine (D71N) has been shown to prevent activation of the M_1_ receptor [15]. This residue is neither part of the agonist binding site nor the receptor-G-protein interface. It is supposed that the D71N mutation disrupts intramolecular hydrogen bond network and prevents the receptor from gaining an active conformation. Its effect on suppressing receptor activation is confirmed in Fig. 5 (upper right). Although D71N receptors cannot be activated by carbachol they still display both high- and low-affinity sites for carbachol with the same proportion as control wild-type M_1_ receptors (Fig. 6, full and open circles). Thus, an active conformation of the receptor is not a prerequisite for receptor-G protein pre-coupling. These data are in perfect fit with a report by Quin et al. [24] published during completion of this manuscript that M_3_ receptors form inactive complexes with G_q_ G-proteins.

In contrast, mutations that interfere with receptor signaling by being directed against the receptor G-protein interface do affect pre-coupling. Mutation of aspartate 122 in the conserved E/DRY-motif at the intracellular edge of the third transmembrane domain to asparagine (D122N) has been shown to reduce the potency of muscarinic agonists [13]. Measurements of the association rate of GTPγS shows that at D122N receptors GTPγS binding under basal conditions (in the absence of agonist) is accelerated and that carbachol has smaller effect on GTPγS association rate than at wild-type M_1_ receptors (Fig. 5, lower left). Meanwhile, the proportion of low-affinity sites for carbachol is increased in D122N receptors in comparison with control (Fig. 6). Most likely, increased basal activity of D122N results in more activated G-proteins and thus more uncoupled receptors in membrane preparations. Crystal structure of complex of opsin and C-terminus of G-protein α-subunit revealed that arginine in the conserved E/DRY-motif at the intracellular edge of the third transmembrane domain interacts directly with the α-subunit C-terminal cysteine [16]. At M_1_ muscarinic receptors mutation of arginine 123 to asparagine (R123N) blocks activation of G-proteins (Fig. 5, lower right). In accordance with the ternary complex model of GPCRs [21], R123N receptors (uncoupled from G-proteins) display only low-affinity for carbachol (Fig. 6, triangles).

It is worth noting that carbachol can activate all three classes of G-proteins at both M_1_ and M_2_ receptors (Fig. 1) and M_1_ receptors pre-couple both to preferential G_q/11_ and non-preferential G_i/o_ G-proteins. In contrast, M_2_ receptors pre-couple only to preferential G_i/o_ G-proteins. G_i/o_ are the major class of G-proteins in membranes from CHO cells, representing almost half of all G-proteins. To exclude the possibility that M_2_ receptors do not pre-couple with G_q/11_ G-proteins due to competition with preferential G_i/o_ G-proteins, we attenuated the expression of G_i/o_ α-subunits by siRNA to one quarter, making G_i/o_ G-proteins the least abundant class in CHO membranes. Such reduction in expression of G_i/o_ G-proteins diminishes the efficacy of carbachol in activating these preferential G-proteins to a level lower than at any of non-preferential G-proteins (Fig. 7). It also reduced its potency (Table 7 vs. Table 1). On the other hand, the potency of carbachol to stimulate GTPγS binding increases at non-preferential G_s/olf_ and G_q/11_ G-proteins, demonstrating competition among G-proteins for M_2_ receptors [25]. In concert, the proportion of low-affinity sites increases and the effect of the anti-G_i/o_ antibody is reduced (Fig. 8, cf. Table 4 and Table 8). Again, these findings indicate the presence of a lower proportion of high-affinity receptor/G-protein complexes. However, the anti-G_q/11_ antibody has no effect on the proportion of low-affinity sites even after such reduction in the expression of G_i/o_ G-proteins. This suggests that the lack of pre-coupling of G_q/11_ G-proteins with M_2_ receptors is not due to competition with G_i/o_ G-proteins.

In summary, we show that muscarinic receptors pre-couple with their preferential class of G-proteins in the absence of an agonist. In contrast to the M_1_ and M_3_ receptors that pre-couple both with preferential G_q/11_ and non-preferential G_i/o_ G-proteins, the M_2_ and M_4_ receptors pre-couple only with their preferential G_i/o_ G-proteins. Lack of pre-coupling of the M_2_ and M_4_ receptors to G_q/11_ G-proteins is not due to competition with preferential G_i/o_ G-proteins. None of the four subtypes of muscarinic receptors pre-couples to G_s/olf_ G-proteins. Thus, the mode of coupling of a given subtype of muscarinic receptors is governed by a combination of the receptor subtype and the class of G-protein. Advanced instrumental methods like fluorescence resonance energy transfer (FRET) between receptor and G-protein [7] and plasmon surface resonance [5] were developed to monitor kinetics of receptor G-protein interactions. Although these methods give better picture of receptor G-protein interaction, our simple method, that can only detect pre-coupling, does not require recombinant systems like FRET-based methods nor reconstituted systems like plasmon surface resonance methods and can be easily applied *ex vivo*, e.g. to tissues of experimental animals.

## Materials and Methods

### Materials

The radioligands [^3^H]-N-methylscopolamine chloride ([^3^H]NMS), guanosine-5′-γ[^35^S]thiotriphosphate ([^35^S]GTPγS), and anti-rabbit IgG-coated scintillation proximity beads were from Amersham (UK). Rabbit polyclonal antibodies against C-terminus of G-protein (G_i/o_, C-10, and G_s/olf_, C-18) were from Santa Cruz Biotechnology (Santa Cruz, CA). Carbamoylcholine chloride (carbachol), dithiotreitol, ethylendiaminotetraacetic acid (EDTA), guanosine-5′-biphosphate sodium salt (GDP), guanosine-5′-[γ-thio]triphosphate tetralithium salt (GTPγS), N-methylscopolamine bromide (NMS), and pilocarpine hydrochloride were from Sigma (St. Louis, MO). Oxotremorine sesquifumarate was from RBI (Natick, MA) and Nonidet P-40 was from USB Corporation (Cleveland, OH). Furfuryltrimethylammonium bromide (furmethide) was kindly donated by Dr. Shelkovnikov (University of St. Petersburg). Small interfering RNA (siRNA) was designed and synthesized by Ambion/Applied Biosystems, Czech Republic.

### Cell culture and membrane preparation

Chinese hamster ovary cells stably transfected with the human M_1_ to M_4_ muscarinic receptor genes (CHO cells) were kindly donated by Prof. T.I.Bonner (National Institutes of Health, Bethesda, MD). Cell cultures and crude membranes were prepared as described previously [18]. Briefly, cells were grown to confluency in 75 cm^2^ flasks in Dulbecco's modified Eagle's medium supplemented with 10% fetal bovine serum. Two million of cells were subcultured to 100 mm Petri dishes. Medium was supplemented with 5 mM butyrate for the last 24 hours of cultivation to increase receptor expression. Cells were detached by mild trypsinization on day 5 after subculture. Detached cells were washed twice in 50 ml of phosphate-buffered saline and 3 min centrifugation at 250 x g. Washed cells were suspended in 20 ml of ice-cold incubation medium (100 mM NaCl, 20 mM Na-HEPES, 10 mM MgCl_2_; pH = 7.4) supplemented with 10 mM EDTA and homogenized on ice by two 30 sec strokes using Polytron homogenizer (Ultra-Turrax; Janke & Kunkel GmbH & Co. KG, IKA-Labortechnik, Staufen, Germany) with a 30-sec pause between strokes. Cell homogenates were centrifuged for 30 min at 30,000 x g. Supernatants were discarded, pellets resuspended in fresh incubation medium and centrifuged again. Resulting membrane pellets were kept at −20°C until assayed within 10 weeks at a maximum.

### Attenuation of expression of G_i/o_ G-proteins

Expression of Gi/o G-proteins α-subunits was attenuated by si-RNAs of following sequences (5′->3′ sense): G_o_, GGC UCC AAC ACC UAU GAA Gtt; G_i1_, CCU CAA CAA AAG AAA GGA Ctt; G_i2_, CCU CCA UCA UCC UCU UCC Utt; G_i3_, GGG AGU GAC AGC AAU UAU Ctt. Cells were treated with complexes of all 4 siRNAs and lipofectamine 48 hours prior to experiment. Final concentrations were 50 nM for each siRNA and 0.5 vol. % for lipofectamine.

### Preparation of new stable cell lines

New stable CHO cell lines expressing wild-type M_1_ and mutant M_1_ receptors have been prepared. Coding sequence of wild-type human M_1_ muscarinic receptor (in expression vector pcDNA ver. 3.2, cDNA resource center, University of Missouri-Rolla, MO, USA) was mutated by PCR and mismatch primers using Qiagene QuickChange kit. Mutations were verified by sequencing of complete receptor coding sequence. Then CHO-K1 cells were transfected with either original M_1_-pcDNA or mutated plasmid using Lipofectamine 2000 (Lipofectamie 10 µl/ml, DNA 0.5 µg/ml). After 48 hours geneticine was added to cultivation medium to final concentration of 800 µg/ml. After selection, the concentration of geneticine was lowered to 50 µg/ml and maintained during cultivation.

### Equilibrium radioligand binding experiments

All radioligand binding experiments were optimized and carried out as described earlier [20]. Briefly, membranes were incubated in 96-well plates at 30 ^o^C in the incubation medium described above that was supplemented with freshly prepared dithiothreitol at a final concentration of 1 mM. Incubation volume was 200 µl or 800 µl for [^3^H]NMS saturation experiments. Approximately 30 and 10 µg of membrane proteins per sample were used for [^3^H]NMS and [^35^S]GTPγS binding, respectively. N-methylscopolamine binding was measured directly in saturation experiments using six concentrations (30 pM to 1000 pM) of [^3^H]NMS for 1 hour. Depletion of radioligand was smaller than 20% for the lowest concentration. For calculations, radioligand concentrations were corrected for depletion. Agonist binding was determined in competition experiments with 1 nM [^3^H]NMS. Membranes were first preincubated 60 min with agonists and IgG antibodies against C-terminus of α-subunits of G-proteins, if applicable, and then incubated with [^3^H]NMS for additional 180 min. Final dilution of antibodies was 1∶200 for G_i/o_ and G_s/olf_ and 1∶500 for G_q/11_. Nonspecific binding was determined in the presence of 10 µM NMS. Agonist stimulated [^35^S]GTPγS binding was measured in a final volume of 200 µl of incubation medium with 200 pM (M_1_ or M_3_ receptors) or 500 pM (M_2_ or M_4_ receptors) of [^35^S]GTPγS and 5 µM (M_1_ or M_3_ receptors) or 50 µM (M_2_ or M_4_ receptors) GDP for 20 min at 30°C after 60 min preincubation with GDP and agonist. Nonspecific binding was determined in the presence of 1 µM unlabeled GTPγS. Incubations were terminated by filtration through Whatman GF/F glass fiber filters (Whatman) using a Tomtech Mach III cell harvester (Perkin Elmer, USA). Filters were dried in vacuum for 1 h while heated at 60°C and then solid scintillator Meltilex A was melted on filters (105°C, 90 s) using a hot plate. The filters were cooled and counted in Wallac Microbeta scintillation counter.

### Scintillation proximity assay

In case of scintillation proximity assay, incubation with [^35^S]GTPγS as described above was terminated by membrane solubilization by the addition of 20 µl of 10% Nonidet P-40. After 20 min 10 µl of individual primary antibodies against C-termini of G-protein α-subunits were added and incubation was continued for 1 h. The final dilution was 1∶500 in case of anti-G_i/o_-α and anti-G_s/olf_-α antibodies and 1∶1000 in case of the anti-G_q/11_-α antibody. One batch of anti-rabbit IgG-coated scintillation beads was diluted in 20 ml of incubation medium and 50 µl of the suspension was added to each well for 3 h. Then plates were spun for 15 min at 1,000 x g and counted using the scintillation proximity assay protocol in a Wallac Microbeta scintillation counter.

### Data analysis

In general binding data were analyzed as described previously [20]. Data were preprocessed by Open Office version 3.2 (www.openoffice.org) and subsequently analyzed by Grace version 5.1 (plazma-gate.weizman.ac.il) and statistic package R version 2.13 (www.r-project.org) on Scientific Linux version 6 distribution of GNU/Linux.

The following equations were fitted to data:

Saturation of radioligand binding
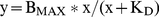
(1)y, binding of radioligand at free concentration of radioligand x; B_MAX_, maximum binding capacity; K_D_, equilibrium dissociation constant.

Concentration-response 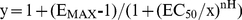
(2)y, radioactivity in the presence of agonist at concentration x normalized to radioactivity in the absence of agonist; E_MAX_, maximal increase by agonist; EC_50_, concentration of agonist producing 50% of maximal effect; nH, Hill coefficient.

Interference of agonist with [^3^H]NMS
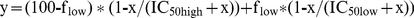
(3)y, binding of radioligand at a concentration of displacer x normalized to binding in the absence of displacer; f_low_, percentage of low affinity sites; IC_50high_, concentration causing 50% decrease in binding to high affinity sites; IC_50low_, concentration causing 50% decrease in binding to low affinity sites. Equilibrium dissociation constant of displacer (K_I_) was calculated according to Cheng and Prusoff [26].

Rate of association
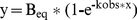
(4)y, binding of radioligand at a time x; B_eq_, equilibrium binding; k_obs_, observed rate of association.

## References

[1] Kroeze WK, Sheffler DJ, Roth BL (2003). G-protein-coupled receptors at a glance.. J Cell Sci.

[2] Overington JP, Al-Lazikani B, Hopkins AL (2006). How many drug targets are there?.. Nat Rev Drug Discov.

[3] Hein P, Bünemann M (2009). Coupling mode of receptors and G proteins.. Naunyn Schmiedebergs Arch Pharmacol.

[4] Shea LD, Neubig RR, Linderman JJ (2000). Timing is everything the role of kinetics in G protein activation.. Life Sci.

[5] Dell'orco D, Koch K (2011). A dynamic scaffolding mechanism for rhodopsin and transducin interaction in vertebrate vision.. Biochem J.

[6] Okuma Y, Reisine T (1992). Immunoprecipitation of alpha 2a-adrenergic receptor-GTP-binding protein complexes using GTP-binding protein selective antisera. Changes in receptor/GTP-binding protein interaction following agonist binding.. J Biol Chem.

[7] Lachance M, Ethier N, Wolbring G, Schnetkamp PP, Hébert TE (1999). Stable association of G proteins with beta 2AR is independent of the state of receptor activation.. Cell Signal.

[8] Hynes TR, Mervine SM, Yost EA, Sabo JL, Berlot CH (2004). Live cell imaging of Gs and the beta2-adrenergic receptor demonstrates that both alphas and beta1gamma7 internalize upon stimulation and exhibit similar trafficking patterns that differ from that of the beta2-adrenergic receptor.. J Biol Chem.

[9] Hein P, Frank M, Hoffmann C, Lohse MJ, Bünemann M (2005). Dynamics of receptor/G protein coupling in living cells.. EMBO J.

[10] Michal P, Rudajev V, El-Fakahany EE, Dolezal V (2009). Membrane cholesterol content influences binding properties of muscarinic M2 receptors and differentially impacts activation of second messenger pathways.. Eur J Pharmacol.

[11] Bonner TI (1989). The molecular basis of muscarinic receptor diversity.. Trends Neurosci.

[12] Nobles M, Benians A, Tinker A (2005). Heterotrimeric G proteins precouple with G protein-coupled receptors in living cells.. Proc Natl Acad Sci U S A.

[13] Michal P, El-Fakahany EE, Dolezal V (2007). Muscarinic M2 receptors directly activate Gq/11 and Gs G-proteins.. J Pharmacol Exp Ther.

[14] Jakubík J, Janíčková H, El-Fakahany EE, Doležal V (2011). Negative cooperativity in binding of muscarinic receptor agonists and GDP as a measure of agonist efficacy.. Br J Pharmacol.

[15] Fraser CM, Wang CD, Robinson DA, Gocayne JD, Venter JC (1989). Site-directed mutagenesis of m1 muscarinic acetylcholine receptors: conserved aspartic acids play important roles in receptor function.. Mol Pharmacol.

[16] Scheerer P, Park JH, Hildebrand PW, Kim YJ, Krauss N (2008). Crystal structure of opsin in its G-protein-interacting conformation.. Nature.

[17] Zhu SZ, Wang SZ, Hu J, el-Fakahany EE (1994). An arginine residue conserved in most G protein-coupled receptors is essential for the function of the m1 muscarinic receptor.. Mol Pharmacol.

[18] Oldham WM, Hamm HE (2008). Heterotrimeric G protein activation by G-protein-coupled receptors.. Nat Rev Mol Cell Biol.

[19] Jakubík J, Bačáková L, Lisá V, el-Fakahany EE, Tuček S (1996). Activation of muscarinic acetylcholine receptors via their allosteric binding sites.. Proc Natl Acad Sci U S A.

[20] Jakubík J, El-Fakahany EE, Doležal V (2006). Differences in kinetics of xanomeline binding and selectivity of activation of G proteins at M(1) and M(2) muscarinic acetylcholine receptors.. Mol Pharmacol.

[21] De Lean A, Stadel JM, Lefkowitz RJ (1980). A ternary complex model explains the agonist-specific binding properties of the adenylate cyclase-coupled beta-adrenergic receptor.. J Biol Chem.

[22] Gilchrist A, Mazzoni MR, Dineen B, Dice A, Linden J (1998). Antagonists of the receptor-G protein interface block Gi-coupled signal transduction.. J Biol Chem.

[23] Sykes DA, Dowling MR, Charlton SJ (2009). Exploring the mechanism of agonist efficacy: a relationship between efficacy and agonist dissociation rate at the muscarinic M3 receptor.. Mol Pharmacol.

[24] Qin K, Dong C, Wu G, Lambert NA (2011). Inactive-state preassembly of G(q)-coupled receptors and G(q) heterotrimers.. Nat Chem Biol.

[25] Kukkonen JP, Näsman J, Akerman KE (2001). Modelling of promiscuous receptor-Gi/Gs-protein coupling and effector response.. Trends Pharmacol Sci.

[26] Cheng Y, Prusoff WH (1973). Relationship between the inhibition constant (K1) and the concentration of inhibitor which causes 50 per cent inhibition (I50) of an enzymatic reaction.. Biochem Pharmacol.

